# Methods and Measures Used to Evaluate Patient-Operated Mobile Health Interventions: Scoping Literature Review

**DOI:** 10.2196/16814

**Published:** 2020-04-30

**Authors:** Meghan Bradway, Elia Gabarron, Monika Johansen, Paolo Zanaboni, Patricia Jardim, Ragnar Joakimsen, Louise Pape-Haugaard, Eirik Årsand

**Affiliations:** 1 Norwegian Centre for E-health Research University Hospital of North Norway Tromsø Norway; 2 Department of Clinical Medicine Faculty of Health Science University of Tromsø The Arctic University of Norway Tromsø Norway; 3 Telemedicine and eHealth Research Group Department of Clinical Medicine University of Tromsø The Arctic University of Norway Tromsø Norway; 4 Norwegian Institute of Public Health Oslo Norway; 5 Tromsø Endocrine Research Group Department of Clinical Medicine University of Tromsø The Arctic University of Norway Tromsø Norway; 6 Division of Internal Medicine University Hospital of North Norway Tromsø Norway; 7 Department of Health Science and Technology Aalborg University Aalborg Denmark

**Keywords:** mobile health, apps, self-management, chronic disease, noncommunicable diseases, interventions, patient-centered approach, patient-operated intervention

## Abstract

**Background:**

Despite the prevalence of mobile health (mHealth) technologies and observations of their impacts on patients’ health, there is still no consensus on how best to evaluate these tools for patient self-management of chronic conditions. Researchers currently do not have guidelines on which qualitative or quantitative factors to measure or how to gather these reliable data.

**Objective:**

This study aimed to document the methods and both qualitative and quantitative measures used to assess mHealth apps and systems intended for use by patients for the self-management of chronic noncommunicable diseases.

**Methods:**

A scoping review was performed, and PubMed, MEDLINE, Google Scholar, and ProQuest Research Library were searched for literature published in English between January 1, 2015, and January 18, 2019. Search terms included combinations of the description of the intention of the intervention (eg, self-efficacy and self-management) and description of the intervention platform (eg, mobile app and sensor). Article selection was based on whether the intervention described a patient with a chronic noncommunicable disease as the primary user of a tool or system that would always be available for self-management. The extracted data included study design, health conditions, participants, intervention type (app or system), methods used, and measured qualitative and quantitative data.

**Results:**

A total of 31 studies met the eligibility criteria. Studies were classified as either those that evaluated mHealth apps (ie, single devices; n=15) or mHealth systems (ie, more than one tool; n=17), and one study evaluated both apps and systems. App interventions mainly targeted mental health conditions (including Post-Traumatic Stress Disorder), followed by diabetes and cardiovascular and heart diseases; among the 17 studies that described mHealth systems, most involved patients diagnosed with cardiovascular and heart disease, followed by diabetes, respiratory disease, mental health conditions, cancer, and multiple illnesses. The most common evaluation method was collection of usage logs (n=21), followed by standardized questionnaires (n=18) and ad-hoc questionnaires (n=13). The most common measure was app interaction (n=19), followed by usability/feasibility (n=17) and patient-reported health data via the app (n=15).

**Conclusions:**

This review demonstrates that health intervention studies are taking advantage of the additional resources that mHealth technologies provide. As mHealth technologies become more prevalent, the call for evidence includes the impacts on patients’ self-efficacy and engagement, in addition to traditional measures. However, considering the unstructured data forms, diverse use, and various platforms of mHealth, it can be challenging to select the right methods and measures to evaluate mHealth technologies. The inclusion of app usage logs, patient-involved methods, and other approaches to determine the impact of mHealth is an important step forward in health intervention research. We hope that this overview will become a catalogue of the possible ways in which mHealth has been and can be integrated into research practice.

## Introduction

### Need for Mobile Health Evaluation

Health research is yet to agree upon a framework for evaluating mobile health (mHealth) interventions. This is especially true for tools, such as apps and wearables, that are intended primarily to aid patients in health self-management. Traditionally, the evaluation of mobile medical devices has been based on clinical evidence, and it can take years to bring these devices to the market. The continuous glucose monitor first came onto the market in 1999, but it was not until 2006 that the next version was available [1]. Similarly, the pulse oximeter struggled for decades to become a standard mobile tool for measuring blood oxygenation [2]. Because there are increasingly easy-to-use patient-operated mHealth technologies available on the market, patients are no longer willing to wait for a lengthy evaluation process. Instead, patients often use apps without assurance of quality or guidance from their health care providers [3].

### Always-Available Self-Management Technologies

Individuals are more empowered to take greater responsibility for their health, and currently, they enthusiastically seek out mHealth apps and other devices for self-management. For chronic conditions in particular, health challenges occur continuously, not just when it is convenient or at a doctor’s office. Technologies for self-management must allow individuals to register and review the measurements that they input into the app or system at any time. Connectivity to devices, such as medical or commercial sensors and wearables, adds to the utility of an app. A report by Research2Guidance [4], an organization that provides market research on digital health, emphasized the central role of patient-operated mHealth apps in the “connectivity landscape” of electronic health technologies [5]. However, their diverse functionalities and intended uses pose great challenges to researchers.

### Challenges of mHealth Evaluation: Single Apps Versus Multiplatform Interventions

The amount of assessment and testing that is necessary for health technology is directly related to its potential risks and benefits [6,7]. For example, medications based on patient-gathered health data are associated with higher health risks than those in patients with type 2 diabetes who seek motivation from an activity tracker for weight management. Although multiplatform (ie, system) interventions serve to increase the benefits (eg, automatic and less burdensome operations), they increase the risks related to data safety, integrity, and reliability [8,9]. Researchers must adapt their approaches, methods, and measures for patient self-management interventions involving single mHealth apps and those involving multiplatform systems.

### Evaluation Framework: Coverage

There are two main categories of mobile medical or mHealth devices associated with the amount of oversight health authorities will show; those that are “actively regulated” and those that fall under “enforcement discretion.” These categories are described in the 2015 Guidance for Industry and Food and Drug Administration Staff [10] and are echoed in the updated 2019 Guidance [11] and included in the terms of The European Economic Area Certification (CE) Mark [12]. Devices that are actively regulated are required to undergo an evaluation and meet security and effectiveness standards for use in health care. On the other hand, many patient-operated technologies fall under “enforcement discretion,” and they pose less risk to patient safety and health. For individuals aiming to assess the usefulness or safety of these technologies, there are no evaluation frameworks or guidelines to follow. The year 2015 marked a relevant change in the mHealth arena, which we are still exploring today (connectivity between different device types, development on different platforms, and marked focus on mHealth integration into clinical practice) [13].

Although there have been many strategies [14-17] for the evaluation of this subset of mHealth (eg, National Institute for Health and Care Excellence [18]), there is no agreement about which qualitative or quantitative measures should be addressed or how they should be evaluated [19]. Evaluation frameworks, such as the World Health Organization (WHO) mHealth evidence reporting and assessment (mERA) checklist [20], suggest that traditional health research measures and methods are not sufficient. For assessing the comprehensive impacts of such patient-operated mHealth approaches, research needs to look into additional factors. This can be achieved by producing evidence that is relevant for both patients and clinicians.

### Additional Factors for mHealth Evaluation

Although clinical evidence is essential for the evaluation of any health aid, the two major concepts of time and human behavior must also be addressed in mHealth evaluation. As “always available” technologies are being used continuously and uniquely by patients, it is uncertain how much time is needed to produce an effect and what changes in self-management behavior will occur. Traditionally, medical devices rely on established biological knowledge, have fewer alternatives in the market, and do not offer frequent updates. However, patient-operated mHealth approaches require the consideration of patients’ motivation, health beliefs, and resources for self-management. They must also compete with hundreds of other mHealth apps and devices that are continuously developed and updated. In recent years, clinical research has attempted to keep pace with mHealth by employing methods that aim to expedite the research process and produce more tailored knowledge for the field of mHealth [21].

Stakeholders associated with chronic health and care (researchers, individuals, health care providers, and health care authorities) have been calling for evidence related to the personal use of mHealth technologies for many years [22-24]. Regardless of the beneficial or harmful outcomes, we need to know their potential. Without such evidence, people in the health care field will not be able to effectively support and guide individuals in the use of these technologies for health self-management. This evidence must be obtained with appropriate questions and methods.

Recent scoping reviews of mHealth technologies for chronic conditions focused on evidence as it relates to a specific age group [25], the development process [26], or clinical outcomes [27] and not on how the research was performed or which resources were used in the evaluation. The purpose of this scoping review was to identify which methods were used and which qualitative and quantitative data were measured to assess patient-operated mHealth devices for the self-management of chronic noncommunicable diseases (NCDs). As evidence for health authorities and health care providers, quantitative clinical outcomes have historically been considered the primary target for evaluation [28]; however, given the growing trend of mHealth, we included qualitative measures of participants’ use of and experiences with the technology.

### Research Questions

The research questions were as follows: (1) What methods are used to evaluate patient-operated mHealth apps and systems for self-management of chronic NCDs? (2) Which qualitative and quantitative measures are used to evaluate the impact of patient-operated mHealth apps and systems for self-management of chronic NCDs?

## Methods

### Scoping Review Objective

We performed a scoping review to document how researchers have evaluated mHealth interventions for self-management of chronic NCDs. Munn et al [29] stated that scoping reviews are favored over other review types in cases in which researchers are using an evolving set of methods owing to the novelty of the field or where the purpose of the review is to inform future questions about the field. We intended to provide an overview of what methods researchers use and which qualitative and quantitative measures were adopted to evaluate mHealth self-management interventions. This review reports information according to the Preferred Reporting Items for Systematic review and Meta-Analyses extension for Scoping Reviews (PRISMA-ScR) checklist (Multimedia Appendix 1).

### Search Strategy and Databases

The scope of the search and definitions of mHealth were discussed among the coauthors (MB, EG, EÅ, and MJ). The databases searched for scientific literature were PubMed, MEDLINE, Google Scholar, and ProQuest Research Library. PubMed and MEDLINE were both included because PubMed includes citations that are not yet indexed in MEDLINE [30]. We searched for articles published in English between January 1, 2015, and January 18, 2019, which were related to the evaluation of patient-operated mHealth interventions for self-management of chronic NCDs. The search string included key terms describing the intervention’s intended use (ie, self-efficacy, self-assessment, self-management, or self-monitoring) and the intervention’s platform (ie, mobile phones, wearables, sensors, or apps). The full search string was used for titles and abstracts, and the format was adapted to the database being searched (Multimedia Appendix 2).

Medical Subject Headings (MeSH) terms were not considered because our search included articles published recently, which may contain terminology that has not yet been indexed within the MeSH database. The identified abstracts and titles were collected in EndNote [31] and then uploaded into Rayyan [32], an online “library systematic review service” that allows researchers to collaborate on the organization, inclusion, and exclusion of articles for literature review.

### Eligibility Criteria

We aimed to include research efforts that may have addressed new guidelines for mobile medical devices. Within our broad search criteria for low-risk mHealth apps and systems, articles were eligible for inclusion if they described low-risk technologies consistent with the FDA and CE Markings’ description of mobile medical devices under “enforcement discretion” [10-12]. Multimedia Appendix 3 describes the specificities of this subcategory.

A preliminary search was performed, and a random selection of 10 articles was reviewed for inclusion or exclusion by two authors (MB and EG). Refinements were made to the review criteria.

For this review, we included studies that evaluated interventions involving (1) mHealth technologies for chronic NCDs, including the primary NCDs listed by the WHO [33] (ie, diabetes, cancer, cardiovascular diseases, chronic respiratory diseases, and chronic mental health conditions); (2) mHealth technologies for self-management (tasks which a person must perform in order to manage the symptoms, treatment, physical and psychosocial consequences, and lifestyle changes inherent in living with a chronic condition, and efficacious self-management was considered to encompass the ability to monitor one’s condition and to affect the cognitive, behavioral, and emotional responses necessary to maintain a satisfactory quality of life) [34]; and (3) mHealth technologies that allow the patient to choose which measures to register and review.

The details of the inclusion and exclusion criteria are described in Multimedia Appendix 4, and they were used during the main review search.

### Data Extraction and Synthesis

After removing duplicate articles, reviews, and protocol articles without evaluation results, two authors (MB and PJ) independently screened the titles and abstracts for eligibility according to the inclusion and exclusion criteria. In case of disagreement regarding eligibility, another author (EG) was called to join the discussion until an agreement was reached. Author MB reviewed the full-text articles and performed data extraction.

The identified studies were classified as either those that evaluated mHealth apps or mHealth systems. Interventions that included a single app were grouped as mHealth apps, whereas those that included services or devices connected to a central app were grouped as mHealth systems. In this way, we could more clearly assess the different approaches taken by researchers when addressing the various impacts of these two mHealth intervention types.

#### Abilities of Studies to Produce Results

For both groups, one author (MB) assessed whether a study was able to produce the evidence that it aimed to obtain, using the selected methods. This was performed by comparing the objectives as stated by the authors of the identified articles to the methods and reported results. The studies were judged according to their ability to produce the information, and the findings were reported as yes, yes and more than expected, no, and cannot tell. The results of these comparisons are detailed in Multimedia Appendix 5.

## Results

### Overview

**Figure 1:**
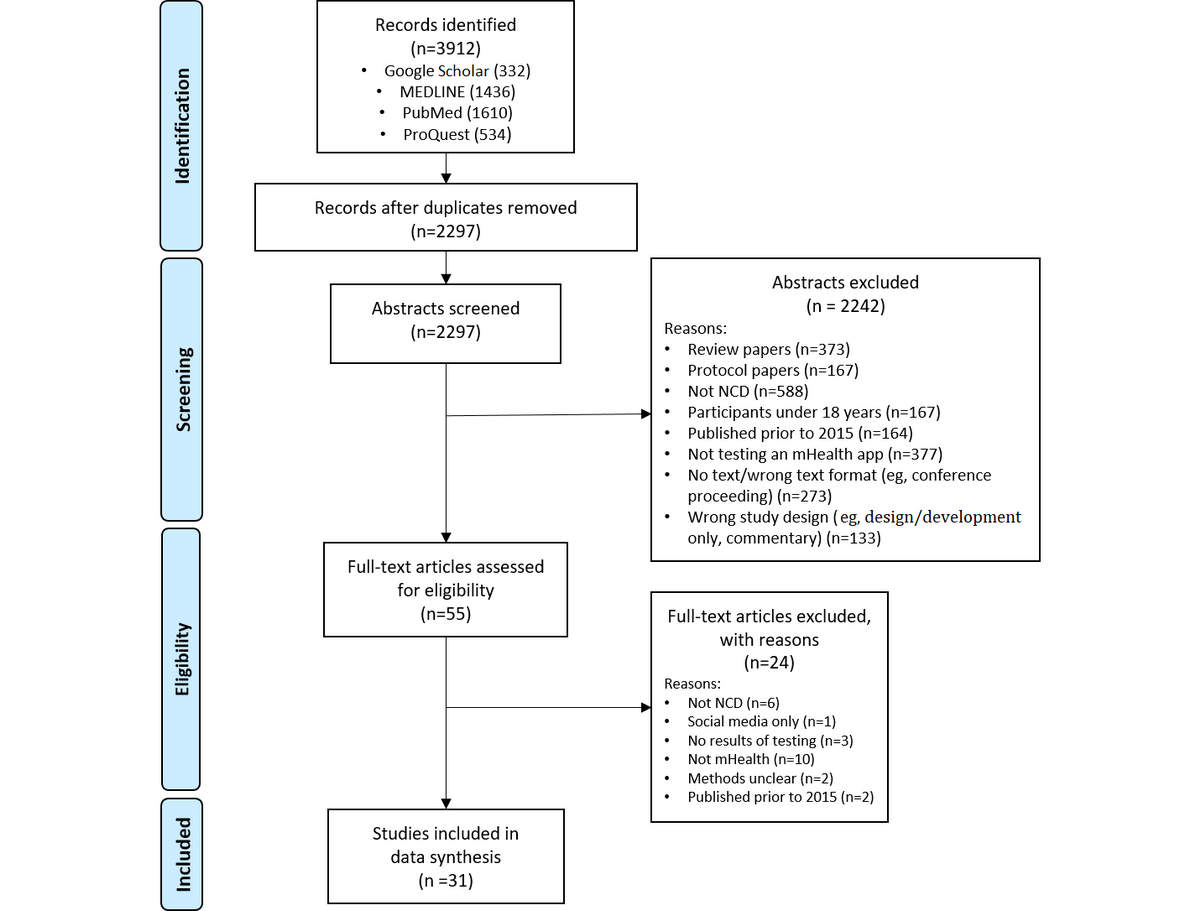
Flow diagram illustrating the selection of studies for inclusion in data synthesis. NCD: noncommunicable disease.

Among 3912 records identified by the search criteria, we reviewed 55 full-text articles and included 31 studies for data extraction and synthesis. Figure 1 illustrates the process of identifying the relevant articles for inclusion in data extraction.

### Summary of Studies: Apps Versus Systems

Among the 31 studies chosen for data extraction, 15 were categorized as those that evaluated mHealth apps and 17 were categorized as those that evaluated mHealth systems. One study evaluated both apps and systems [35] and was therefore included in both categories. General information about the selected studies that evaluated mHealth apps are summarized in Table 1 [35-49] and those that evaluated mHealth systems are summarized in Table 2 [35,50-65].

**Table 1:** Information about the studies that evaluated mHealth apps.

Reference	App name	Year	Country	Study design	Duration	Health condition	Patient participants	Health care provider and caregiver participants	Intended secondary users
[36]	Diet and Activity Tracker (iDAT)	2015	Singapore	Prospective study	8 weeks	Type 2 diabetes	Patients (n=84)	N/A^a^	N/A
[37]	Diabetes Notepad	2015	Korea	Cross-sectional study	Single evaluation	Diabetes	Patients (n=90)	N/A	N/A
[38]	Personal Life-chart app	2015	Germany	Prospective study	72 weeks	Bipolar disorder	Patients (n=54)	N/A	N/A
[39]	HeartKeeper	2015	USA	Cross-sectional study	Single evaluation	Heart diseases	Patients (n=24) and researchers	N/A	N/A
[40]	HeartKeeper	2016	Spain	Retrospective study	36 weeks	Heart diseases	Patients (n=32)	N/A	N/A
[41]	PTSD Coach	2015	USA	Retrospective study	Duration of availability of the app on app stores	Post-traumatic stress disorder	Current users (n=156)	N/A	N/A
[42]	PTSD Coach	2015	USA	RCT^b^	16 weeks	Post-traumatic stress disorder	Patients (n=10)	Health care providers (n=3)	Health care providers
[43]	PTSD Coach	2016	USA	RCT	4 weeks	Post-traumatic stress disorder	Patients (n=49)	N/A	N/A
[44]	PTSD Coach	2017	USA	RCT	24 weeks	Post-traumatic stress disorder	Patients (n=120)	N/A	N/A
[45]	Hypertension management app (HMA)	2016	Korea	—^c^	Single event evaluation	Hypertension	Patients (n=38)	Nurses (n=3) and experts (n=5)	N/A
[35]^d^	Multiple commercial apps for heart failure	2016	USA	Cross-sectional study	Single evaluation	Heart failure	Apps (n=34)	N/A	Family, friends, and health care providers (not all apps)
[46]	Multiple commercial apps (n=11)	2016	USA	Cross-sectional study	Single evaluation	Multiple	Patients (n=20)	Caregivers (n=9)	N/A
[47]	I-IMR intervention	2017	USA	Cross-sectional study	Single evaluation	Serious mental health conditions^e^	Patients (n=10)	N/A	N/A
[48]	Serenita	2017	Israel	Prospective study	16 weeks	Type 2 diabetes	Patients (n=7)	Health care providers	N/A
[49]	Sinasprite database	2018	USA	Retrospective study	6 weeks	Depression and anxiety	Patients (n=34)	N/A	N/A

^a^N/A: not applicable.

^b^RCT: randomized controlled trial.

^c^Not available.

^d^Study evaluated both apps and systems and therefore will appear in both categories.

^e^Combination of cardiovascular disease, obesity, diabetes, high blood pressure, high cholesterol, osteoporosis, gastroesophageal reflux disease, osteoarthritis, chronic obstructive pulmonary disease, congestive heart failure, coronary artery disease, and bipolar disorder, major depressive disorder, schizophrenia, or schizoaffective disorder [47].

**Table 2:** Information about the studies that evaluated mHealth systems.

Reference	Intervention name	Year	Country	Study design	Duration	Health condition	Participants	Intended secondary users	Others involved in the intervention	Medical device included (Y/N)	Other devices included
[50]	SUPPORT-HF Study	2015	UK	Cross-sectional study	45 weeks	Heart failure	Patients (n=26)	Health care providers	Health care providers and informal care givers	Y	Blood pressure monitor, weight scales, and pulse oximeter
[51]	—^a^	2015	USA	Cross-sectional study	Single evaluation	Diabetes	Patients (n=87) and health care providers (n=5)	Health care providers	Health care providers	Y	Glucose meter
[52]	Multiple commercial technologies for activity tracking	2015	USA	Prospective study	80-100 days (mean 12.5 weeks)	Serious mental health condition^b^	Patients (n=10)	Health care providers and peers (optional)	N/A^c^	N	Wearable activity monitoring devices
[53]	Diabetes Diary app	2015	Norway	Prospective study	2 weeks	Type 1 diabetes	Patients (n=6)	N/A	N/A	Y	Smart-watch app and glucose meter
[54]	Diabetes Diary app	2015	Norway	RCT^d^	23 weeks	Type 1 diabetes	Patients (n=30)	N/A	N/A	Y	Glucose meter
[55]	Diabetes Diary app	2016	Norway	RCT	48 weeks	Type 2 diabetes	Patients (n=151)	Health care providers	N/A	Y	Glucose meter
[56]	SnuCare	2016	Korea	Prospective study	8 weeks	Asthma	Patients (n=44)	N/A	Research team	Y	Peak flow meter
[57]	HealthyCircles Platform	2016	USA	RCT	24 weeks	Hypertension	Patients (n=52)	Health care providers	Health care providers	Y	Withings blood pressure monitor
[58]	Multiple commercial technologies for activity tracking	2016	USA	Prospective study	24 weeks	Serious mental health condition^b^	Patients (n=11)	N/A	N/A	N	Fitbit Zip
[35]^e^	Multiple commercial apps for heart failure	2016	USA	Cross-sectional study	Single evaluation	Stroke	Apps (n=34)	Family, friends, and health care providers (not all apps)	N/A	N	Y
[59]	Electronic Patient Reported Outcome tool (ePRO)	2016	Canada	Prospective study	4 weeks	Multiple	Patients (n=8) and health care providers (n=6)	Health care providers	Health care providers	N	N
[60]	STARFISH	2016	UK	Prospective study	6 weeks	Stroke	Patients (n=23)	Peers (automatic)	N/A	N	ActivPAL™ activity monitor
[61]	HeartMapp	2016	USA	Cross-sectional study	Single evaluation	Heart failure	Patients (n=25) and health care providers (n=12)	Health care providers	Health care providers	Y	Zephyr Bioharness or Biopatch
[62]	EDGE digital health system	2017	UK	RCT	48 weeks	Chronic obstructive pulmonary disease	Patients (n=110) and research nurses (n=2)	Health care providers (automatic)	Informal care givers	N	N
[63]	IBGStar Diabetes Manager Application	2017	Germany	Prospective study	12 weeks	Diabetes	Patients (n=51)	N/A	N/A	Y	iBGStar blood glucose meter
[64]	MyHeart	2017	USA	Prospective study	24 weeks	Heart failure	Patients (n=8) and nurses	Nurses (automatic)	Nurses	Y	Weight scale, blood pressure monitor, and glucose meter
[65]	—	2018	UK	Cross-sectional study	4 weeks	Cancer	Patients (n=23)	Peers and health care providers	N/A	N	N

^a^Not available.

^b^Schizophrenia spectrum disorder, bipolar disorder, or major depressive disorder [52,58].

^c^N/A: not applicable.

^d^RCT: randomized controlled trial.

^e^Study evaluated both apps and systems and therefore will appear in both categories.

App interventions mainly targeted mental health conditions (n=7), followed by diabetes (n=3) and cardiovascular and heart diseases (n=4), with one study evaluating multiple apps that were used to self-manage multiple health conditions (Table 1).

Patients were included in all studies, and the studies had between 3 and 156 participants (median 36, IQR 15-87, maximum 156). The exception was one study in which only researchers evaluated patient-operated apps according to Google recommendations and quality standards [35,39]. Although studies tested single apps intended to be used primarily by patients, two studies also explored the impact of patients sharing their collected data with health care providers [35,42].

Six studies utilized single evaluations, either through a cross-sectional design [35,37,39,45-47] or an analytic service to analyze data available through the app store [41]. The remaining studies evaluated the impacts of app use over time, lasting between 4 and 72 weeks, with a mean period of 22.75 weeks (median 16 weeks, IQR 6-36, maximum 72). Of these, four utilized prospective study designs, three were randomized controlled trials (RCTs), and two used a retrospective design.

Among the 17 studies that described mHealth systems, most involved patients diagnosed with cardiovascular and heart disease (n=6), followed by diabetes (n=5), respiratory disease (n=2), mental health conditions (n=2), cancer (n=1), and multiple illnesses (n=1; Table 2).

As with mHealth app studies, all system studies, except one [35], involved patients. The 16 studies had between 6 and 151 patients (median 30, IQR 14.5-51.5, maximum 151), with eight studies involving health care providers. In these cases, health care providers either provided input on the suitability of an app for patient use or reviewed patient-gathered data during consultations.

In 12 studies, patients were required to share data (n=6) [50,51,57,60,62,64] or encouraged to share data (n=6) [35,53,55,59,61,65] with their health care providers or peers as part of the study. Data were also collected and transmitted to the main app by medical devices [50,51,53-57,61,63,64] and commercial wearables [35,52,53,58,60], demonstrating the prevalence of connectivity in modern mHealth systems.

Few studies (n=3) used single evaluations. RCTs (n=4) lasted longer (35.75 weeks on average) than cross-sectional studies (mean 24.5 weeks, n=2) and prospective studies (mean 12.93 weeks, n=7). Overall system evaluations lasted a mean of 20.32 weeks, which is very close to that for app interventions, but with a higher median number of 23 weeks.

### Methods and Measures

Most studies included a combination of qualitative and quantitative methods of evaluation. Evaluation of usage logs was the most commonly adopted method (21 studies), followed by standardized questionnaires (17 studies; Table 3). Only two studies adopted quality guidelines to evaluate mHealth interventions; the Mobile Application Rating Scale was used to evaluate multiple apps [35], and compliance with Google standards for Android systems, in addition to other approaches, was used to evaluate the HeartKeeper app [39].

**Table 3:** Categories of methods used to evaluate mHealth interventions.

Methods (adopted approaches)	Studies that evaluated mHealth apps	Studies that evaluated mHealth systems
Evaluation of usage logs	[36,38,40-42,44,48,49]	[50,52,54,56-59,62-64]
Standardized questionnaires	[35-39,41-45,48,49]	[35,55-57,60,64]
Ad-hoc questionnaires	[36,37,40,42-44,47]	[51,53,55-58,61-63]
Interviews	[40,45,46]	[50,52,58,59,65]
Clinical outcomes	[36,48]	[54-56,63,64]
Open feedback (ie, oral or written)	[35,41,43,45]	[35,53,62]
Collection of additional device data (eg, medical device data)	N/A^a^	[54,56,57,60,62,64]
Field study and observation	[46,47]	[61,65]
Focus groups	N/A	[59,64]
Observational tests (in a lab setting)	[45,47]	N/A
Quality guidelines	[35,39]	[35]
Medical record entries	[42]	[63]
Attendance (intervention assigned activities/meetings)	[42,48]	N/A
Download count	[41]	N/A

^a^N/A: not applicable.

Among the 14 ad-hoc questionnaires used, four were developed according to concepts or questions from standardized questionnaires [47,58,61,62]. Similarly, two studies included interviews, where the interview guides were based on standardized questionnaires [40,45]. Some standardized questionnaires were used in more than one study. Multimedia Appendix 6 lists these questionnaires and illustrates the combination of questionnaires used in each study. Compared with traditional medical device testing, relatively few studies included information gathered from medical record entries (n=2), clinical outcomes (n=9), or observational tests (n=2).

Of note, some studies inferred more information from usage logs than the count and type of app interactions and patient-gathered data. For example, Triantafyllidis et al [50] interpreted information from the evaluation of usage logs on the usability of the device and participants’ engagement in the study. The complete set of the types of data that were measured and collected by the mHealth app and system intervention studies are listed in Table 4.

**Table 4:** Categories of qualitative and quantitative data that were measured to evaluate mHealth interventions.

Types of data measured	Studies that evaluated mHealth apps	Studies that evaluated mHealth systems
Interactions (via app)	[36,37,40-42,44,45,49]	[50,52,53,56-59,62-65]
Usability/feasibility	[35,37,39-42,45,47]	[35,52,53,56,58,59,61,62,65]
Patient-gathered self-management data (via app)	[36-38,41,45,49]	[50,54,55,57,59,62-64]
Efficacy/effectiveness	[35-37,40,42,43,45,48]	[35,50,51,53,56,58,59,64,65]
Physical well-being	[36,40,42,48]	[54-57,60,62-64]
Perceptions, opinions, and suggestions	[35,40,41,45-47]	[35,51-53,58,64,65]
Intervention experiences	[39,41,46,47]	[50,52,58,59,64,65]
Psychological well-being	[38,41,42,44,49]	[55,60,62]
Patient-reported health	[40-44]	[56,63]
Self-efficacy	[36,44,47,49]	[55,57,61]
Engagement/motivation in self-management	[36,41]	[50,52,56,63]
Health care utilization and impact	[42]	[56,59,62-64]
Task performance	[45-47]	[50,61,65]
Study engagement	[35,41,42,48,49]	[35]
Patient-reported app use	[43,44]	[53,58,59]
Patient-reported self-management	[36,37]	[52,57,60]
Quality of life	[48]	[55,56,60,64]
App features and quality	[35,39,41,47]	[35]
Efficiency	N/A^a^	[62,65]
Security	[39]	[51]
Lifestyle	[48]	N/A

^a^N/A: not applicable.

Although a single method can often provide information regarding more than one measure, over one-third of the studies in this review used more than one method to collect information on one type of measure [40,42,45,48,50,55-60]. For example, two studies used both the collection of additional device data and clinical outcomes to report physical well-being [54,64]. Multimedia Appendix 7 includes a description of which measures were produced by each method. Several of the studies collected information on twice as many types of data measured as methods used to collect them (n=9) [35,41,44,49,58-60,65], with two studies collecting three [51,52] and one collecting four [39] times the number of types of data measured as methods used to collect them. Only one study used four methods to evaluate the most unique data types that were measured (n=10) by utilizing information resources that mHealth technologies make available (eg, automatically collected data from current users in the Android app store) [41].

Conversely, measures can be reported using more than one method. For example, usability/feasibility was the most common measure (22 times in 17 studies), followed by efficacy/effectiveness (20 times in 16 studies), interactions (via app; 19 times in 19 studies), physical well-being (18 times in 13 studies), and patient-gathered self-management data (via app; 15 times in 14 studies; Multimedia Appendix 7).

The study by Possemato et al [42] described the only app intervention that measured health care utilization and impact from these methods*.* Kim et al [56], Alnosayan et al [64], and Sieber et al [63] described system interventions that measured health care utilization or impact (ie, hospitalizations reported by participating health care providers and hospitalizations recorded retroactively). The remaining studies (n=5) collected information regarding physical well-being from clinical outcomes measured by researchers or health care providers during follow-up [36,48,54,55,61].

More comprehensive mapping of methods and measures revealed that the methods that were used to produce the most diverse set of data were, as expected, interviews (n=9), standardized questionnaires (n=16), and study-specific questionnaires (n=13; Multimedia Appendix 7). However, evaluation of usage logs produced nearly as many different types of measures (n=8).

### Objectives and Methods Versus Results

A comparison of the study objectives with the results demonstrated that 30 of the 31 studies reported the results that they intended. One study reported all but one of the intended results described in the original objectives (ie, whether the reviewed apps and systems had been previously validated) [35]. Ten studies reported more than they anticipated, some of which included the assessment of app [42,48] and system [50] usage patterns, as well as comparisons with other outcomes [41,44]. Other unforeseen outcomes included the accuracy of the app’s knowledge base, as evaluated by nurses [45]; usability according to patients’ performance of predetermined tasks with the app [47]; usability of connected devices in an mHealth system [53]; health care utilization [56]; and patient-reported symptoms [63]. Two studies stated that the objective was to develop mHealth systems; however, their outcomes also included evaluation results [50,51]. None of the studies phrased their goals as research questions and some reported what they intended, but the objective was not explicitly stated or detailed [40,63]. For example, Velardo et al [62] stated their intention to evaluate their intervention at scale. However, it was not clear how they intended to “evaluate” their intervention.

## Discussion

### Principal Findings

We identified 31 studies that described evaluations of mHealth apps or systems, with one describing evaluation of both intervention types [35]. Our findings show that studies relied mostly upon more continuous measures. Except for the collection of additional device data used by system interventions but not app interventions, there were no significant differences between apps and systems with regard to their ability to produce the intended outcomes, health conditions, or types of methods or measures used within the studies. Overall, medical record entries [42], attendance of meetings or activities assigned by the intervention [63], and download count [41] were the least used methods for gathering information about an intervention’s impact on patients and providers. On the other hand, evaluation of usage logs [36,38,40-42,44,48-50,52,54,56-59,62-64] and standardized questionnaires [35-39,41-45,48,49,55-57,60,64] were the most commonly used methods. These two approaches (ie, one traditional and one mHealth) were also commonly used together in the same studies, demonstrating that mHealth is supplementing, not replacing, traditional research approaches.

### mHealth Trends Versus Methods and Measures Used

Although clinical integration of mHealth technologies is on the rise, only two studies described app interventions that were meant to be used by secondary users (ie, health care providers and family and friends) [35,42], with three involving health care providers in the evaluation process [42,45,48]. Despite the focus on data safety and security, as well as patient privacy, as described by the new General Data Protection Regulation [66] and established FDA [10,11] and CE marking [12] expectations for health-related technologies, only two studies included measures regarding security [39,51].

### Need to Reassess Evaluation Standards

Health evaluation studies are meant to produce evidence and understanding of how various interventions could affect patients and providers in real-world health care settings. Traditionally, studies have been classified within a hierarchy based on their designs, methods, and measures used to evaluate health interventions [67]. Health professionals consider high-level studies to be those that use rigorous and strict study designs, such as RCTs [68]. These studies provide an objective and quantitative understanding of how an intervention would influence patient clinical health measures, cost, or health care resource use [69]. On the other hand, low-level studies are often those that rely upon subjective and flexible study designs (eg, qualitative studies of participants’ perception of the intervention or its impact on their lifestyle) [70].

### Challenges of Quality Assessment

Health intervention researchers are not given instructions or guidance about how to evaluate these mHealth apps or which additional evidence is needed to determine their comprehensive impacts on patients and providers. The recent addition of connected technologies, such as wearables and sensors, has introduced even more factors to the evaluation context. Interventions now vary from recording exercise, to decision support for patient self-management, to providing evidence of a patients’ actions for health care providers, to review from a variety of data sources. Because of these new information sources, we cannot always anticipate all of the impacts of these diverse networks of mHealth self-management technologies. For example, 10 studies did not intend to obtain results related to certain factors, such as usage logs and patient-reported outcomes [41,42,44,50,53,63].

The assessment of a study’s success, validity, or quality presents another challenge to traditional research practice. mHealth resources consist of factors that make standard quality assessments inconclusive for intervention studies. For example, identifying patterns of patient self-management habits and progress describes the impact of an mHealth intervention on a patient’s behavior. However, the analysis of usage logs, as a measure of intervention effectiveness, patient engagement, or self-management practices, has been minimally investigated as an appropriate method. As demonstrated by some of the reviewed articles, usage logs, download counts, and online ratings of apps were interpreted as indications of patient engagement, self-management behavior, intervention reach [41], effectiveness, and intervention utility [40] or feasibility.

### Comparing Objectives and Results to Determine Successful Use of Methods

As opposed to completing a formal quality assessment, we chose to determine whether a study was able to produce the evidence that it aimed to provide, using selected methods. Some studies that performed usage log analysis were able to produce more information than they anticipated. Possemato et al [42] stated their intention to assess the fidelity of the PTSD Coach intervention by comparing health care utilization and health outcomes between those who used the app with and without clinical support. They were able to provide evidence for the effectiveness and fidelity of the intervention among health care providers, symptoms, and clinical health parameters from questionnaires. Moreover, they provided evidence for participants’ patterns of intervention use from usage logs. Thereby, they were able to discuss the relationship between health care provider involvement and reinforced use of the app, as patients may have felt more accountable for using the app to self-manage their post-traumatic stress disorder.

Among the 31 studies identified, one did not obtain all of the intended information (missing one of the intended outcomes) [35] and one was found to be inconclusive [53]. We found that it was challenging to determine the specific objective of a study when objectives were not stated as such or when they were vague. This made it difficult to determine if a study was successful in the use of its selected methods and study design to reach its goals. For example, Velardo et al [62] stated that they intended to evaluate the EDGE digital health system intervention at scale; however, they did not state how they intended to do so or provide a research question that they intended to answer. Sieber et al [63] did not state the objective of their study. Instead, they stated simply what was done (ie, investigated the effects of usage profiles on hemoglobin A1c). Without a stated objective, we are unable to judge the reliability of intervention studies, whether it be through standard traditional means or an alternative approach. Clear objectives must be included in order to validate mHealth resources as trustworthy and relevant measures for evaluating mHealth interventions.

### Relevance

mHealth must work for health care providers as well as patients. Patients are more engaged in their health, and they incorporate mHealth into their self-management. Thus, patients are aware of and can even influence how an mHealth intervention should or could be used to influence the kind of impact that is relevant for them. Understanding the potential risks and benefits of patient-operated mHealth requires more continuous evidence of not only technical and clinical outcomes but also personal and psychological impacts. This review demonstrates, through the use of such measures as mHealth interactions and patient-gathered data via an app, that we as researchers have the resources at our disposal and are beginning to use them.

A 2016 study by Pham et al [71] called for alternative or additional methods and measures for mHealth clinical trials that address the additional needs of mHealth. As most mHealth technologies for chronic health self-management are intended to be always available and continuously used by the patient, research questions, approaches, and designs need to reflect the real-world situations in which patients use these apps and systems.

Several studies within the presented scoping review demonstrated an attempt to meet this call by including more flexibility in their intervention design. For example, the EDGE digital health system [62], PTSD Coach app [42,43], and HeartKeeper app [40] made the patient the “decision maker” by allowing the patient to choose which data are relevant for them to gather and share with their health care providers. Further, two studies focused on reporting that patient engagement improved as a result of using mHealth apps [36,52]. User engagement is a necessity for the success of any intervention. It is paramount to consider patients’ intentions when using these apps outside of the clinic; we should deem an app’s ability to engage patients with their health as necessary as clinical evidence. There are individuals who do not choose to manage their chronic illnesses at all, for example, those deemed “hard to reach,” who may benefit from merely acknowledging their health challenge by using an app primarily for education, without the expectation of performing complicated and time-consuming self-management. Therefore, when judging the success, usefulness, or potential benefit of an evaluated mHealth intervention, there should be less of a hierarchical gap between clinical health change or improvement and patients’ experiences and change in self-efficacy.

### Limitations

We believe our review covers most of the articles that were published during the established period and dealt with mHealth interventions for chronic conditions. This review reported on patient-operated mHealth self-management and did not include other potentially relevant interventions, such as SMS-based interventions.

We chose to focus on self-management of chronic NCDs, as defined by the WHO, in addition to severe mental health conditions, according to the demand for solutions from two fields (the medical system and public app development market) [4,13,33,72]. As such, these health cases represented the most potential for including state-of-the-art technology studies, with chronically ill people consistently being the leading market. However, exclusion of preventive treatments and other chronic health challenges (eg, musculoskeletal diseases) may have excluded a large proportion of cases that both involve the use of self-management options and represent a relevant portion of the chronic disease burden for individuals and health care systems worldwide [73]. As such, this noninclusion may have omitted conditions that could have provided relevant insights into methods and measures used to assess motivational, educational, and empowering mHealth technologies for self-management.

Because we did not collect data on reported results for this scoping review and did not perform a systematic methodological quality assessment, we cannot comment on the usefulness or effectiveness of the mHealth app and system interventions presented in these studies.

### Conclusion

Researchers are now using several mHealth resources to evaluate mHealth interventions for patient self-management of select NCDs. This is evident as studies relied mostly on more continuous measures, including usage logs [36,38,40-42,44,48-50,52,54,56-59,62-64] and patient-collected data from medical devices [54,56,57,60,62,64], in addition to pre-post measures, such as clinical health measures [36,40,48,54-56,63,64] and standardized questionnaires [35-39,41-45,48,49,55-57,60,64]. In doing so, they evaluated the health status, engagement, and feasibility of mHealth apps and systems. In this review, which focused on mHealth, we found that only 20% of the included studies relied solely on traditional study designs (eg, RCTs) and methods that measure only pre- and postintervention health changes. The findings illustrate that the tradition of focusing on “clinical effectiveness, cost-effectiveness, and safety” [74] or health-related quality of life and the use of health care resources [75] is not being replaced, but is instead being expanded by taking advantage of additional resources that mHealth provides to evaluate interventions.

There is still no clear standard for the evaluation of mHealth interventions for patient self-management of chronic conditions. However, because mHealth presents additional challenges, needs, and resources to the field of health intervention research, we have the opportunity to expand and maintain our relevance to patients, providers, and health authorities. mHealth provides new types of information that we can and should gather to determine the impact of the interventions.

The presented results demonstrate that health studies have started to take advantage of additional mHealth resources, such as app usage logs and other patient-involved research methods, to determine the comprehensive impacts of mHealth on patients and other stakeholders. We are able to not only answer questions, such as which tasks patients choose to perform during interventions that may affect their clinical outcomes, but also say more about the relevance of mHealth for various types of users. This is essential in health intervention research, as the call for evidence on mHealth continues to push for not only traditional clinical health measures but also impacts on patients’ self-efficacy and engagement. We believe that to achieve a compromise between the rigidity of traditional quality standards and the push for more patient-relevant outcomes, the definition of quality or meaningful impact, as well as available and appropriate evidence should be reassessed.

## Appendix

Multimedia Appendix 1 PRISMA-ScR checklist.

Multimedia Appendix 2 Search strategy.

Multimedia Appendix 3 Scope of included technologies.

Multimedia Appendix 4 Inclusion and exclusion criteria by category.

Multimedia Appendix 5 Comparison of study objectives to reported results.

Multimedia Appendix 6 List of questionnaires and scales used in mHealth intervention studies.

Multimedia Appendix 7 Mapping of measures to methods.

## Abbreviations

MeSH: Medical Subject Headings
mHealth: mobile health
NCD: noncommunicable disease
RCT: randomized controlled trial
WHO: World Health Organization
