# Supplementary material for: Methods and Measures Used to Evaluate Patient-Operated Mobile Health Interventions: Scoping Literature Review
Source: JMIR Mhealth Uhealth. 2020 Apr 30;8(4):e16814. doi: 10.2196/16814 (PMC7226051; doi:10.2196/16814)

## **Appendix 2.** Search strategy

**Table 1.** The number of articles found as the result of the literature search strategies specific to each database.


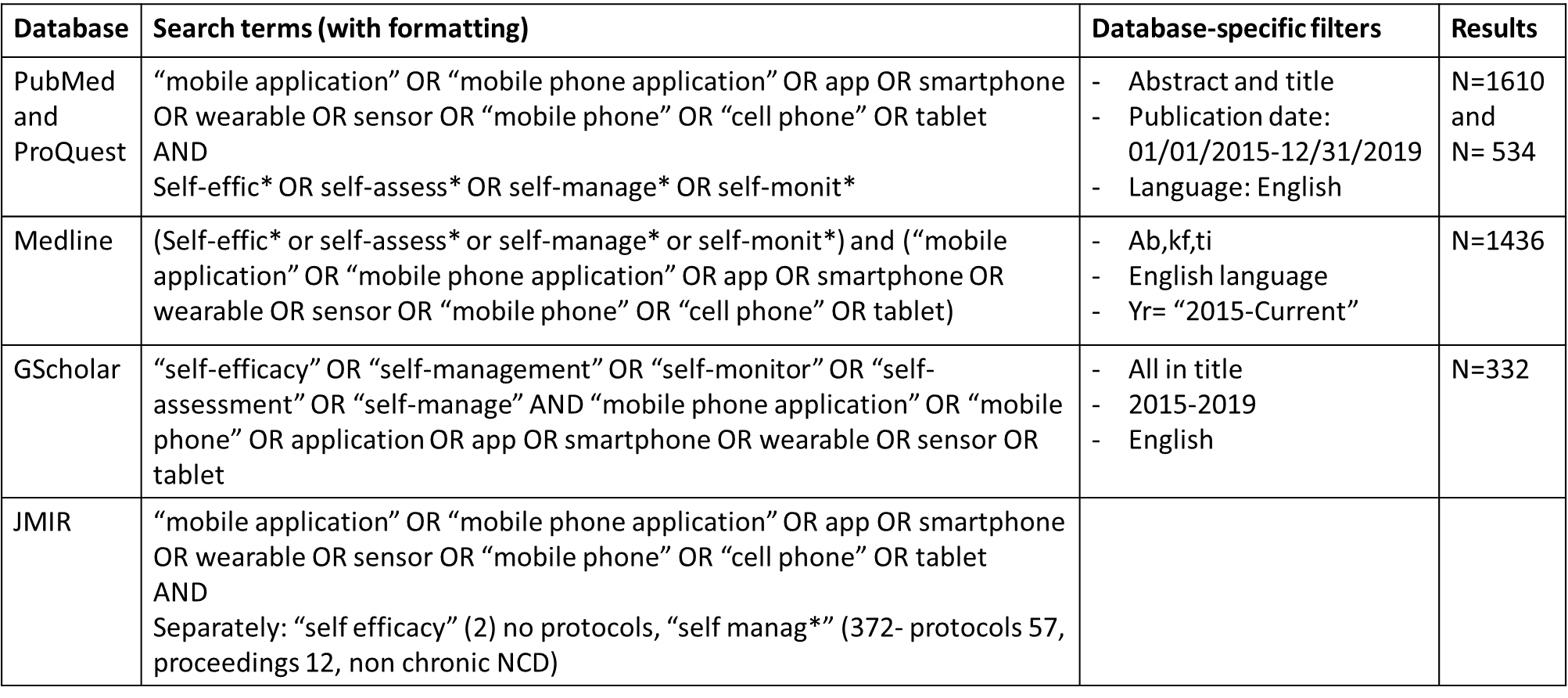

Supplement: Multimedia Appendix 2 [file mhealth_v8i4e16814_app2.docx]
