# Supplementary material for: Methods and Measures Used to Evaluate Patient-Operated Mobile Health Interventions: Scoping Literature Review
Source: JMIR Mhealth Uhealth. 2020 Apr 30;8(4):e16814. doi: 10.2196/16814 (PMC7226051; doi:10.2196/16814)
Supplement: Multimedia Appendix 3 [file mhealth_v8i4e16814_app3.docx]

# **Appendix 3.** Explanation of included mHealth interventions

In 2015, the FDA released a guidance document describing which mHealth devices and apps would be considered under their purview. The scope of included mHealth interventions for chronic NCD self-management is based upon those technologies that do not yet have guidelines for their assessment, i.e. those under the FDA’s “enforcement discretion” as described in *Mobile Medical Applications:* *Guidance for Industry and Food and Drug Administration Staff* [1].

The guidance emphasizes that the level of evaluation and independent review should be commensurate with the risk posed, and encourages manufacturers to use continuous monitoring to understand and modify software based on real-world performance [2].

These, therefore, exist under the FDA’s purview, i.e. “premarket review and post-market regulatory requirements, including but not limited to registration and listing and premarket notification requirements”. However, there are no specific instructions for which methods and measures should be used. Here we provide an overview of which methods and measures research groups have chosen to use, assumedly, in response to these updated guidelines.


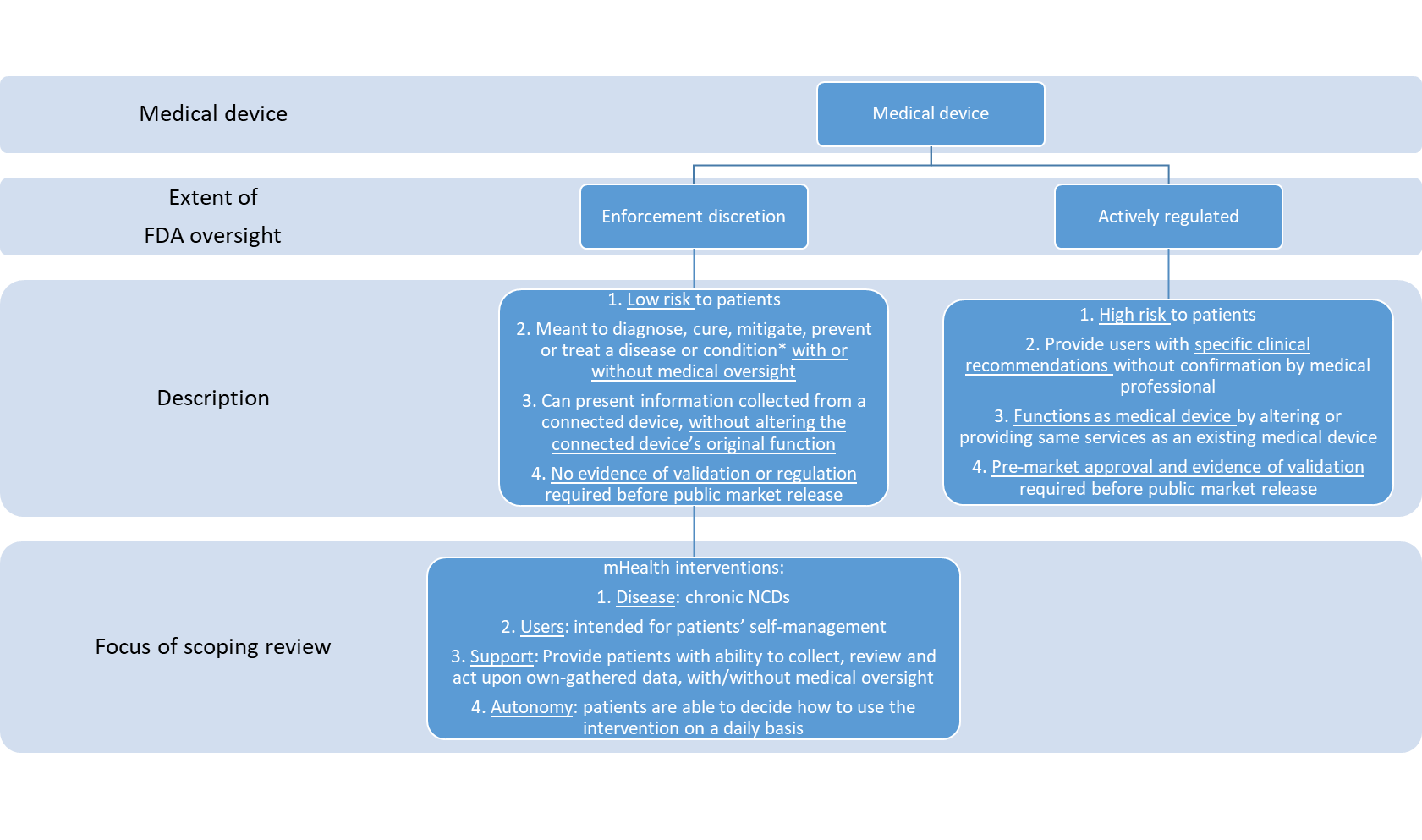


*Paraphrased from the FDA guidance document [1]

**Figure 1.** Flow/hierarchy identifying the scope of the review

## References

1. Administraion USFD. Mobile Medical Applications. Guidance for Industry and Food and Drug Administration Staff. Rockville, MD, USA: U.S. Food & Drug Administration; 2015.

2. Software as a Medical Device (SaMD). fda.gov: U.S. Food & Drug Administration; 2018 [cited 2019]; Available from: <https://www.fda.gov/medical-devices/digital-health/software-medical-device-samd>.
