# Supplementary material for: Methods and Measures Used to Evaluate Patient-Operated Mobile Health Interventions: Scoping Literature Review
Source: JMIR Mhealth Uhealth. 2020 Apr 30;8(4):e16814. doi: 10.2196/16814 (PMC7226051; doi:10.2196/16814)
Supplement: Multimedia Appendix 4 [file mhealth_v8i4e16814_app4.docx]

# **Appendix 4.** Inclusion and exclusion criteria

**Table 1.** Inclusion and exclusion criteria by category

| **Category** | **Inclusion criteria** | **Exclusion criteria** |
| --- | --- | --- |
| Intended use | - Primarily meant for the patient to use for self-management of chronic NCD *(family and health care professionals may be included, but as secondary users)* - Available at all times, i.e. also without internet connection | - Primarily monitoring or coaching of patients’ health by health care professional - Automatically generated clinical recommendations, e.g. by app, system or health care professional - Data collection for research or clinical use only - Disease prevention |
| Study population | - Adults with a diagnosed chronic NCD - Health care providers, or others, involved in the testing of the intervention | - Inclusion of individuals below 18 years of age - Acute diseases, e.g. lasting less than 1 year [69] - Those at risk for, i.e. not diagnosed with, a chronic NCD |
| Platform | - Information is “always available” on a mobile device, i.e. smartphone, tablet, or wearable - Technology must allow individuals to register and review measurements that patients themselves input into the platform, both on- and off-line, with the capability to upload and store said data when internet connectivity is re-established. - Platforms are classified as either i) an individual app, on any of the aforementioned devices, or ii) a system, i.e. more than one device and/or app inter-communicating to collect and review data - Intended to enable primarily patients to autonomously review and react to collected data | - Classified/certified as medical devices - Computer based technologies only - Web-dependent technologies - SMS based intervention |
| Evaluation | - Assessment, validation, evaluation, testing, validity, feasibility of an mHealth intervention | - Design and/or development without testing - Not primary research, i.e. reviews - No results of evaluating an mHealth technology, e.g. protocols, commentaries, general surveys etc. |
