# Supplementary material for: Methods and Measures Used to Evaluate Patient-Operated Mobile Health Interventions: Scoping Literature Review
Source: JMIR Mhealth Uhealth. 2020 Apr 30;8(4):e16814. doi: 10.2196/16814 (PMC7226051; doi:10.2196/16814)
Supplement: Multimedia Appendix 5 [file mhealth_v8i4e16814_app5.docx]

# **Appendix 5.** Critical appraisal

Here we present the integrity or success of results based upon comparison to stated research questions, objectives or aims.

**Table 1.** Results of comparing reported outcomes to stated objectives of app intervention studies.

| **Ref[s]** | **Intervention name** | **Objective, aims, purpose, intention related to evaluation of the intervention** | **Methods** *(used for evaluation)* | **Reported results*** | **Did they report what they aimed?**  *(Y,N, more than expected (Y+), Can't tell)* |
| --- | --- | --- | --- | --- | --- |
| [36] | Diet and Activity Tracker” (iDAT) | ID usage patterns and associated patient characteristics based on collected data | - Clinical measures - Evaluation of usage logs - Standardized questionnaires - Ad-hoc questionnaires | - Emergent groups of patients’ usage patterns (“Latent Trajectory Groups”) - Predictors of group membership (diet, exercise motivation, standardized questionnaire scores, and sex) - In discussion: Able to distinguish usage patterns of the app into 3 groups | **Y** |
| [37] | Diabetes Notepad | To obtain data about usefulness of app (including whether diabetes SM activities improved and to what extent the activities improved according to satisfaction) | - Standardized questionnaires - Ad-hoc questionnaires | - User satisfaction (changes in clinical course of diabetes, structure of app, ease of use, willing to recommend to others, willing to continue using the app) - Changes in activities after use of the application (standardized questionnaire) | **Y** |
| [38] | Personal Life-chart app | Investigate correlation between impairment of social function vs. score of depression symptoms (clinician rated), and mania rating both in depressed and hypomanic states. ALSO to examine correlation between self-reported mood and clinician ratings | - Evaluation of usage logs - Standardized questionnaires | - Symptoms (standardized questionnaires) - Correlation between data entered in life-chart data and symptom scores and mood | **Y** |
| [39] | Heartkeeper | Use 2 different tools to assess the quality based on compliance with Google guidelines and users’ quality of experience | - Quality guidelines - Standardized questionnaires | - Compliance with android guidelines (Google) - Quality of experience (from standardized questionnaire) | **Y** |
| [40] |  | Summary of what is evaluated in the paper: usage, utility of app evaluated for different groups in different areas  *Note: aims and purposes were mostly stated throughout the text in relation to each measure and method, not the overall project* | - Evaluation of usage logs - Field study and observation - Interview - Study-specific questionnaire | - Effectiveness (change in health measures, usability, patient opinions, patient use of app) - Utility (experienced symptoms, performance of self-management activities before and after intervention use) - Differences between sex and age based on measures of utility | **Y** *(objective or aims* *not explicitly stated in the introduction)* |
| [41] | PTSD Coach | Characterize reach, use and impact of intervention, how users engaged with the app, reception and impact of app in users of general pop, potential differences between android and iOS | - Evaluation of usage logs - Download count - Open feedback - Standardized questionnaires | - Reach of intervention (downloads, active users, descriptions of users) - Use of intervention (user retention, usage patterns, reviews) - Reception and impact (perception of meaningful outcomes) - Usability (standardized questionnaire) - Reported symptoms (entered into app) - Differences between iOS and Android - Also reported: self-reported symptoms vs. use patterns | **Y+** |
| [42] |  | Evaluate feasibility and potential effectiveness of the app with and without clinical support (also to inform future RCT, for both research procedures and intervention conditions), gather prelim data to investigate if the clinician support improves severity and healthcare utilization compared to the app alone and by how much more they experienced improvement with the clinician app | - Attendance to intervention assigned activities/meetings - Evaluation of usage logs - Medical records - Standardized questionnaires - Ad-hoc questionnaires | - Retention - Fidelity amongst clinicians (Ad-hoc questionnaire) - Symptoms and clinical health (standardized questionnaires) - Post-intervention referral acceptance and treatment - Comparison of effectiveness of the app alone vs. with clinician support on healthcare usage and health outcomes - Also reported: app usage | **Y+** |
| [43] |  | Primary: feasibility (usage and barriers to use), acceptability (whether users found it useful), potential efficacy (changes in self-reported symptoms) of app in reducing symptoms and relationship between them | - Open feedback - Standardized questionnaires - Ad-hoc questionnaires | - Feasibility (study-specific questionnaire) - Acceptability via self-reported assessment of helpfulness of app vs. paper materials (Ad-hoc questionnaire and open feedback) - Potential efficacy via symptoms, (standardized questionnaire) | **Y** |
| [44] |  | Assess the efficacy of the intervention: hypothesized that patients randomized to 3months of using the app would achieve greater improvements in symptoms severity, self-efficacy, depression severity, psychosocial functioning vs. control AND it would last three months after | - Evaluation of usage logs - Standardized questionnaires - Ad-hoc questionnaire | - Symptoms and health outcomes (standardized questionnaires) - Self-efficacy (Ad-hoc questionnaire) - Also reported: app use (and correlations between self-reported app use and changes in health outcomes) | **Y+** |
| [45] | Hypertension management app (HMA) | Evaluate intervention’s effectiveness by measuring perceived usefulness, user satisfaction and medication adherence | - Interviews - Lab tests - Open feedback - Standardized questionnaires | - Perceived usefulness and medication adherence (standardized questionnaire and interviews) - User satisfaction for relevant functions (Ad-hoc questionnaire and interviews) - Also reported: Accuracy of knowledge base and mobile heuristics evaluation by nurses (lab tests) | **Y+** |
| [35]* | - | Identify apps, describe characteristics, identify if any apps have been rigorously testing, rate apps based on MARS, IMS institute and Heart Failure society guidelines and scales | - Quality guidelines - Open feedback - Standardized questionnaires | - Identified apps’ characteristics and functionalities - MARS scale scores - IMS scores - Compliance with guidelines - Overall app quality - NOT included: statement of whether or not apps had been rigorously tested before | **N** *(did not present results of whether or not apps had been previously tested)* |
| [46] | Multiple commercial apps (n=11) | Investigate usability of existing apps for variety of conditions in order to facilitate development and tailoring of these | - Field study and observation - Interviews | - Patients’ performance and experienced barriers completing tasks - Experiences, i.e. patients’ frustration, lack of confidence in using these apps, interest in tech to support self-management - Reported in discussion: Suggestions from researchers for how to specifically improve apps based on their experience | **Y** |
| [47] | I-IMR intervention | Describe process of adapting intervention/development *(not analysed as part of this review but was performed in the study),* generalization to others | - Field study and observation - Lab tests - Ad-hoc questionnaires | - *Design* *(not part of this review but it was done)* - Also reported: usability (via Ad-hoc questionnaires), and patients’ performance and experience completing tasks - Reported in discussion*:* generalization | **Y+** |
| [48] | Serenita | Assess the effect of incorporating the app in care (i.e. on weight, BP and glycaemic measures) | - Attendance to intervention assigned activities/meetings - Evaluation of usage logs - Clinical measures - Standardized questionnaires | - Changes in health outcomes - Also reported: adherence to the program and self-management, health and well-being outcomes (standardized questionnaires) - NOT reported: cumulative time on app due to technical errors | **Y+** |
| [49] | Sinasprite database | Evaluate the app in order to assess self-directed engagement and how use of the app impacted self-reported confidence in coping skills and severity of depression and anxiety | - Evaluation of usage logs - Standardized questionnaires | - Symptom and health outcomes (standardized questionnaires) - Relationship between demographics and probability of symptomology - Use of app vs. Probability of symptomology, demographics and self-reported outcomes - Mobile app engagement (usage logs) | **Y** |

* Note that the reported results that appeared in the results sections may be different from the measures described in the methods sections. This is often because authors describe the measures as a category of different reported outcomes, e.g. the measure “usability” can include such specific outcomes as “learnability” or “ease of use”.

**Table 2.** Results of comparing reported outcomes to stated objectives of system intervention studies.

| **Ref[s]** | **Intervention name** | **Objective, aims, purpose, intention related to evaluation of the intervention** | **Methods** *(used for evaluation)* | **Reported results*** | **Did they report what they aimed?**  *(Y,N, more than expected (Y+), Can't tell)* |
| --- | --- | --- | --- | --- | --- |
| [50] | The SUPPORT-HF Study | Design and iteratively move towards an app that meets the needs of patient group  *Note:* u*nder 3.2 of the results, they argue that the evaluation is part of the development process* | - Evaluation of usage logs - Interviews | - *Design iterations and results (not part of this review but it does accomplish the goals stated by the paper)* - Also reported: adherence, engagement, effectiveness, efficiency (usage logs) - NOT reported: satisfaction (via interviews, “will be presented in the final paper”) | **Y+** *(while they argue that the evaluation is part of the iterative development, they do not state it in the intro or methods but only in the results)* |
| [51] | - | To develop and evaluate based on standard protocol and international standard information model to support effective BG management  *Note:* *we will only focus on the reports of evaluation* | - Ad-hoc questionnaires | - Accuracy, usefulness by HCPs - Usage intention by patients and HCPs, reliability and validity (usage intention, effort expectancy, social influence, facilitating condition, perceived risk, voluntariness) | **Y+** *(because the main intention was to describe the development)* |
| [52] | - | Inform future research on effectiveness of interventions, usability and acceptable for target population | - Evaluation of usage logs - Interviews | - Learnability and engagement (usage logs) - Satisfaction, helpfulness, usefulness, interest in future use, feasibility and acceptability | **Y** |
| [53] | Diabetes Diary app | If the concept (SM app that communicates with smartwatch) using Pebble extends the usefulness of the app | - Open feedback - Ad-hoc questionnaires | - Users’ expectations, perceptions, app use and feasibility (appreciated, not-appreciated and desired features) - Also reported: general usability of the smartwatch, not in comparison with app (study-specific survey), and accuracy of the app and design modifications *(not part of evaluation but still reported and not expected to be reported)* | **Y+** |
| [54] |  | Hypothesis: number of out-of-range (OOR) values as measure of variability of BG, combination of OOR events and HbA1c levels provide a more complete picture of diabetes than one alone *(no mention of the intervention)* | - Evaluation of usage logs - Clinical measures - Medical device data | - Identified groups based on clinical health measure - Patient-collected health measures - Groups (based on HbA1c patterns) vs. other outcomes | **Y** *(yet the intervention was not described in* *the introduction or abstract intro sections)* |
| [55] |  | Investigate stages of change for exercise and diet habits using baseline data from users in intervention by examining associations: stages of change vs. PS and D, stages of change vs. behavior and individual characteristics, QoL, SM, depressive symptoms and lifestyle | - Clinical measures - Standardized questionnaires - Ad-hoc questionnaires | - Identified groups based on “Stages of change” (Ad-hoc questionnaire) - Groups vs. self-management, physical and mental health, well-being, lifestyle and symptoms (standardized questionnaires), and health behaviors | **Y** |
| [56] | SnuCare | Explore feasibility and efficacy of app | - Evaluation of usage logs - Clinical measures - Medical device data - Standardized questionnaires | - Feasibility (usage logs) - Efficacy (medical device data, clinical measures, and standardized questionnaires) - Also reported: healthcare utilization | **Y+** |
| [57] | HealthyCircles Platform | Influence of wireless SM program and PAM on health behaviors, medication adherence, BP levels and control of BP in hypertensive patients | - Evaluation of usage logs - Medical device data - Standardized questionnaires - Ad-hoc questionnaires | - Health engagement measure (standardized questionnaire) - Health engagement measure vs. health behaviors (Ad-hoc questionnaire), health measures and adherence - Interaction with intervention vs. outcome variables - Temporal trends of frequency in self-management of health measures | **Y** |
| [58] | - | Explore participants’ experiences, if participants would sustain use over 6-months, usability, satisfaction, as well as obtaining feedback in order to inform design of future clinical trial | - Evaluation of usage logs - Interviews - Ad-hoc questionnaires | - Duration of intervention use - Usability and satisfaction (Ad-hoc questionnaires) - Experience and feedback (reasons for lack of use, difficulties, interest/ engagement in intervention for future use) - Reported in conclusion: Ideas for future studies and their own clinical trials | **Y** |
| [35]** | - | *Note: reported above* | | | |
| [59] | Electronic Patient-Reported Outcome tool (ePRO) | Aims were to 1) determine whether the app was feasible to be used by patients and their HCPs as part of primary care services, and 2) assess usability from perspectives of both user groups | - Evaluation of usage logs - Focus groups - Interviews | - Feasibility: user ad use of technology, task and technology (all methods used) - Usability: efficiency, effectiveness, learnability, satisfaction (all methods used) | **Y** |
| [60] | STARFISH | Potential effectiveness in improving physical activity, sedentary time, walking speed, markers of health and well-being | - Additional device measures - Standardized questionnaires | - Mean number of steps/day - Effects of group and time interaction, health habits/self-management tasks, symptoms, health and well-being measures (standardized questionnaires) | **Y** |
| [61] | HeartMapp | Describe development *(not included in this analysis)*, preliminary assessment of usability and self-confidence of both patients and providers | - Field study and observation - Ad-hoc questionnaires | - Usability by both patients and HCPs: ease of use, problem-solving, accuracy of materials, satisfaction (Ad-hoc questionnaire) - Self-confidence of both patients and HCPs (Ad-hoc questionnaires) - Participants’ task completion | **Y** |
| [62] | EDGE digital health system | Statement of intention: to evaluate our intervention at scale in RCT  *Note: does not explicitly state what is meant by “evaluate” here* | - Evaluation of usage logs - Medical device data - Open feedback - Ad-hoc questionnaires | - Data quality, System usage, performance of the algorithms, patient compliance, learning curve, usage by nurse - Health measures - NOT reported: Symptoms (Ad-hoc questionnaire and usage logs and open feedback) because it was stated by the authors that this was not the focus of this paper | **Y** *(but objective not explicitly stated or detailed)* |
| [63] | IBGStar Diabetes Manager Application | The study investigated the effects of usage profiles on HbA1c  *Note: Did not state objective but instead simply what was done in the study* | - Evaluation of usage logs - Clinical measures - Medical records | - Self-management of health - Changes in health measures - Also reported: symptoms and safety | **Y+** *(but objective not explicitly stated or detailed)* |
| [64] | MyHeart | Understand challenges, provide design considerations for system that could effectively support/features for HF patients after transitioned into home environment, and nurses experiences | - Evaluation of usage logs - Clinical measures - Focus groups - Medical device data - Standardized questionnaires | - Usage patterns - Clinical health changes and healthcare service usage - Usability and well-being (standardized questionnaire) - Patients’ and nurses’ experiences | **Y** |
| [65] | - | Pilot e-health app via usability and feasibility | - Field study and observation - Interviews | - Participants’ task performance - Usability, acceptability, satisfaction, and feasibility | **Y** |

* Note that the reported results that appeared in the results sections may be different from the measures described in the methods sections. This is often because authors describe the measures as a category of different reported outcomes, e.g. the measure “usability” can include such specific outcomes as “learnability” or “ease of use.”
