# Supplementary material for: Methods and Measures Used to Evaluate Patient-Operated Mobile Health Interventions: Scoping Literature Review
Source: JMIR Mhealth Uhealth. 2020 Apr 30;8(4):e16814. doi: 10.2196/16814 (PMC7226051; doi:10.2196/16814)
Supplement: Multimedia Appendix 6 [file mhealth_v8i4e16814_app6.docx]

# **Appendix 6.** List of questionnaires and scales used in mHealth intervention studies

**Table 1.** List of standardized questionnaires and scales (alphabetically) with references to the articles in which they were used.

| **Standardized questionnaires and scales*** | **References** |
| --- | --- |
| Brief inventory of psychosocial functioning (B-IPF) | [44] |
| Coping Self Efficacy Scale (CSE) | [49] |
| Davis’ perceived usefulness and perceived ease-of-use measurement scales (based on) | [45] |
| Diabetes Empowerment Scale-Short Form (DES-SF) | [36] |
| General Anxiety Disorder Scale (GAD-2 and GAD-7) (based on) | [62] |
| General Anxiety Disorder 7-Item (GAD-7) | [49] |
| Godin Leisure-Time Exercise Questionnaire (GLTEQ) | [58] |
| Google standards for Android developers | [39] |
| Health Education Impact Questionnaire (HeiQ) | [55] |
| Heart Failure Society of America (HFSA) guidelines for nonpharmacologic management | [35] |
| IMS Institute for Healthcare Informatics functionality scoring system | [35] |
| Life events checklist | [44] |
| MARS scale | [35] |
| Minnesota Living with Heart Failure Questionnaire (MLHFQ) | [64] |
| Modified Morisky Scale (MMS) | [45] |
| Patient Health Questionnaire Depression Scale 8 (PHQ-8) | [44, 49] |
| Patient Health Questionnaire 8 (PHQ-8) (based on) | [62] |
| Patient health questionnaire-9 (PHQ-9) | [42] |
| Patient health questionnaire (PHQ-2) (based on) | [62] |
| Post Study System Usability Questionnaire (based on) | [47] |
| PTSD Checklist- Civilian (PCL-C) | [41, 43, 44] |
| PTSD checklist- specific (PCL-S) | [41] |
| Quality Adjusted Life Years (based on) | [40] |
| Quality of Experience (QoE) | [39] |
| Quality of life questionnaire (SF-12) | [48] |
| Quality of Life Questionnaire for Adult Korean Asthmatics (QLQAKA) | [57] |
| Recommendations of the “Non-pharmacologic Management and Health Care Maintenance in Patients | [35] |
| Self-efficacy scale (based on) | [61] |
| Short Form 36 Health Survey (V.2) (SF-36) | [55] |
| Subjective Units of Distress Scale (SUDS, or distress thermometer) | [41] |
| Summary of Diabetes Self-Care Activities (SDSCA) | [37] |
| System Usability Scale (based on) | [47] |
| System Usability Scale (SUS) | [64] |
| The Centre for Epidemiologic Studies Depression scale (CES-D) | [55] |
| The Fatigue Severity Scale (FSS) | [60] |
| The Instrumental Activities of Daily Living Scale (IADL) | [60] |
| The Inventory of Depressive Symptomology, clinician rated (IDS-C) | [38] |
| The Patient Activation Measure (PAM) | [57] |
| The Psychological General Well-Being Index (PGWBI) | [60] |
| The Stroke Specific Quality of Life Scale (SS-QOL) | [60] |
| The Ten-Meter Walking Test (10 MWT) | [60] |
| Usability questionnaire of mobile phone apps (based on) | [61] |
| Usefulness, Satisfaction, and Ease questionnaire (based on) | [47, 58, 61] |
| World health organization quality of life (WHO-QOL) - BREF | [42] |
| Young Mania Rating (YMRS) | [38] |

*Standardized questionnaires include those that were used in whole or in part, i.e. as the basis for study-specific questionnaires created by the authors.
