# Supplementary material for: Methods and Measures Used to Evaluate Patient-Operated Mobile Health Interventions: Scoping Literature Review
Source: JMIR Mhealth Uhealth. 2020 Apr 30;8(4):e16814. doi: 10.2196/16814 (PMC7226051; doi:10.2196/16814)
Supplement: Multimedia Appendix 7 [file mhealth_v8i4e16814_app7.docx]

# **Appendix 7.** Mapping of which methods were used to collect which measures

**Table 1.** Qualitative and quantitative measures grouped by the methods that were used to collect them, with references to the articles in which they were used.

| **Methods** | **Measures** | **Refs_20200121** |
| --- | --- | --- |
| **Additional device measures** | Physical well-being | [54, 56, 57, 60, 62, 64] |
| **Attendance to intervention assigned activities/meetings** | Study engagement | [42, 48] |
| **Clinical measures** | Healthcare utilization and impact | [64] |
|  | Patient-reported health | [40] |
|  | Physical well-being | [36, 48, 54-56, 63, 64] |
|  | Psychological well-being | [55] |
|  | Self-efficacy | [55] |
|  | Healthcare utilization and impact | [56] |
| **Evaluation of usage logs** | Efficacy/effectiveness | [40, 59] |
|  | Efficiency | [62] |
|  | Engagement/motivation in self-management | [56] |
|  | Interactions | [36, 40-42, 44, 49, 50, 52, 53, 56, 57, 59, 62-65] |
|  | Patient-gathered self-management data | [36, 38, 41, 49, 50, 54, 57, 59, 62-65] |
|  | Patient-reported health | [56] |
|  | Patient-reported self-management | [36] |
|  | Perceptions, opinions, suggestions | [53] |
|  | Psychological well-being | [42] |
|  | Security | [51] |
|  | Self-efficacy | [51] |
|  | Study engagement | [48-50, 63] |
|  | Usability/Feasibility | [36, 50, 51, 56, 58, 62] |
| **Download count** | Usability/Feasibility | [41] |
| **Field study and observation** | App features and/or quality | [47] |
|  | Efficacy/effectiveness | [65] |
|  | Efficiency | [65] |
|  | Perceptions, opinions, suggestions | [40] |
|  | Self-efficacy | [47] |
|  | Task performance | [46, 61, 65] |
|  | Usability/Feasibility | [40, 47, 50, 65] |
| **Focus groups** | Efficacy/effectiveness | [59] |
|  | Healthcare utilization and impact | [59] |
|  | Intervention experiences | [59, 64] |
|  | Perceptions, opinions, suggestions | [59, 64] |
|  | Usability/Feasibility | [59] |
| **Interviews** | Efficacy/effectiveness | [45] |
|  | Engagement/motivation in self-management | [52] |
|  | Intervention experiences | [46, 47, 50, 52, 58, 59, 65] |
|  | Patient-reported app-use | [58, 59] |
|  | Patient-reported health | [40] |
|  | Patient-reported self-management | [52] |
|  | Perceptions, opinions, suggestions | [45-47, 52, 58, 65] |
|  | Usability/Feasibility | [45, 50, 52] |
| **Lab tests** | Intervention experiences | [47] |
|  | Perceptions, opinions, suggestions | [45, 47] |
|  | Task performance | [45, 47] |
| **Medical records** | Efficacy/effectiveness | [42] |
|  | Healthcare utilization and impact | [42, 63] |
| **Open feedback** | App features and/or quality | [35, 41] |
|  | Healthcare utilization and impact | [62] |
|  | Intervention experiences | [41] [43] |
|  | Patient-reported app use | [43] |
|  | Patient-reported app-use (updated from usability on 20190822) | [43] |
|  | Perceptions, opinions, suggestions | [41, 43, 45] |
|  | Study engagement | [41] |
| **Standardized questionnaires** | App features and/or quality | [35, 39] |
|  | Efficacy/effectiveness | [35, 56, 64] |
|  | Intervention experiences | [39] |
|  | Lifestyle | [48] |
|  | Patient-gathered self-management data | [45] |
|  | Patient-reported app-use | [43, 44] |
|  | Patient-reported health | [41-44] |
|  | Patient-reported self-management | [37, 57, 60] |
|  | Perceptions, opinions, suggestions | [35] |
|  | Physical well-being | [42] |
|  | Psychological well-being | [38, 41, 42, 44, 49, 60] |
|  | Quality of life | [48, 55, 56, 60, 64] |
|  | Security | [39] |
|  | Self-efficacy | [36, 44, 49, 57] |
|  | Study engagement | [35] |
|  | Usability/Feasibility | [35, 39] |
| ***Ad-hoc* questionnaires** | Efficacy/effectiveness | [37, 40] |
|  | Engagement/motivation in self-management | [36] |
|  | Patient-gathered self-management data | [55] |
|  | Patient-reported app-use | [43, 53] |
|  | Patient-reported self-management | [57] |
|  | Perceptions, opinions, suggestions | [51, 53] |
|  | Physical well-being | [40, 55] |
|  | Psychological well-being | [62] |
|  | Self-efficacy | [44, 61] |
|  | Usability/Feasibility | [42, 43, 47, 53, 58, 61] |
